# Performance of Derived Laboratory Biomarkers with Regard to 30-Day Mortality in Kidney Transplant Recipients with COVID-19

**DOI:** 10.3390/life12122068

**Published:** 2022-12-09

**Authors:** Josipa Domjanović, Tea Domjanović Škopinić, Josipa Radić, Mirko Luketin, Ivo Jeličić, Andrija Matetic

**Affiliations:** 1 Department of Nephrology, University Hospital of Split, 21000 Split, Croatia; 2 Department of Cardiology, University Hospital of Split, 21000 Split, Croatia; 3 Department of Internal Medicine, University of Split School of Medicine, 21000 Split, Croatia

**Keywords:** kidney transplant recipients, COVID-19, laboratory-derived biomarkers

## Abstract

There are limited data on the performance of laboratory-derived biomarkers in kidney transplant recipients (KTR) with COVID-19. This observational study enrolled 65 KTR with COVID-19 who were treated at the University Hospital of Split up to March 2022. Laboratory-derived biomarkers (neutrophile-to-lymphocyte (NLR) ratio, platelet-to-lymphocyte ratio, monocyte-to-lymphocyte ratio, De Ritis ratio, C-reactive protein (CRP)-to-albumin ratio, lactate dehydrogenase (LDH)-to-hemoglobin ratio, CRP-to-lymphocyte ratio, red cell distribution width-to-albumin ratio, platelet-to-albumin ratio, D-Dimer-to-albumin ratio, D-Dimer-to-NLR ratio, LDH-to-albumin ratio, and LDH-to-white blood cell (WBC) ratio) were calculated, and their performance with regard to 30-day mortality was determined. Mortality events occurred in 12 patients (18.5%), which was significantly associated with increased De Ritis (HR 3.83, 95% CI 1.57–9.35, *p* = 0.003), CRP-to-albumin (HR 1.36, 95% CI 1.13–1.64, *p* = 0.001), LDH-to-hemoglobin (HR 1.44, 95% CI 1.07–1.92, *p* = 0.015), CRP-to-lymphocyte (HR 1.03, 95% CI 1.01–1.07, *p* = 0.003), D-dimer-to-albumin (HR 4.94, 95% CI 1.38–7.24, *p* = 0.038), LDH-to-albumin (HR 1.20, 95% CI 1.05–1.36, *p* = 0.008), and LDH-to-WBC (HR 1.03 95% CI 1.01–1.05, *p* = 0.024) ratios. Out of these, the best area-under-the-curve (AUC) values were achieved with De Ritis (AUC 0.691), CRP-to-albumin (AUC 0.764), LDH-to-hemoglobin (AUC 0.877), CRP-to-lymphocyte (AUC 0.739), and LDH-to-albumin (AUC 0.827) ratios, while the best discrimination displayed LDH-to-hemoglobin ratio (Harrell’s C 0.808 and Somers’ D 0.616). The overall calibration was satisfactory for all models. Derived laboratory biomarkers such as the de Ritis, CRP-to-albumin, LDH-to-hemoglobin, CRP-to-lymphocyte, and LDH-to-albumin ratios show significant association and discrimination with all-cause mortality in KTR with COVID-19, suggesting its potential risk stratification role.

## 1. Introduction

Novel Coronavirus disease (COVID-19) continues to produce a substantial healthcare burden with immense morbidity and mortality. Solid-organ transplant recipients including kidney transplant recipients (KTR) represent a particularly fragile patient population in the setting of the COVID-19 pandemic [1]. Due to impaired kidney function, chronic immunosuppression, high comorbidity burden, and increased frailty, this population exhibits high patient heterogeneity and high COVID-19 mortality reaching up to 32% [2,3,4].

High needs of KTR with COVID-19 mandate proper risk stratification to improve management and prognosis, but the literature contains limited data. The ImAgeS score is the only tool that was primarily developed for risk stratification in this patient population [5], while other risk scores such as the CROW-65 risk score were also validated in this setting [6]. However, it is unknown whether other potentially useful risk scores, which were primarily investigated in the overall COVID-19 population, could reproduce similar performances in KTR.

Emerging studies showed a promising association of simple derived laboratory biomarkers with prognosis in the overall COVID-19 population. One of the most studied biomarkers is neutrophil-to-lymphocyte ratio (NLR), which showed satisfactory prediction of severe disease and mortality [7,8]. Admission values of the De Ritis ratio, monocyte-to-lymphocyte ratio and D-dimer-to-albumin ratio exhibited association with in-hospital mortality in the general COVID-19 population [9,10,11]. However, the available literature is lacking information about their performance in KTR with COVID-19.

Therefore, this study aimed to evaluate the performance of different biomarkers with regard to 30-day mortality in KTR with COVID-19, including NLR, platelet-to-lymphocyte ratio, monocyte-to-lymphocyte ratio, De Ritis ratio, C-reactive protein (CRP)-to-albumin ratio, lactate dehydrogenase (LDH)-to-hemoglobin ratio, CRP-to-lymphocyte ratio, red cell distribution width (RDW)-to-albumin ratio, platelet-to-albumin ratio, D-Dimer-to-albumin ratio, D-Dimer-to-NLR ratio, LDH-to-albumin ratio, and LDH-to-white blood cell (WBC) ratio.

## 2. Materials and Methods

### 2.1. Ethical and Institutional Considerations

The study was approved by the Medical Research Ethical Committee of the University Hospital of Split, Croatia (No. 2181-147-01/06) and was conducted with all ethical standards and amendments of the Declaration of Helsinki (ISO 14155).

### 2.2. Study Design and Patients

This retrospective observational study enrolled a total of 65 adult KTR with COVID-19 infection in the period from August 2020 to March 2022. The study was conducted at the University Hospital of Split. All enrolled patients had COVID-19, confirmed by using the reverse transcription-polymerase chain reaction (RT-PCR) test from oro- and nasopharyngeal swab.

The initial patient screening included a total of 67 KTR, while 2 KTR were excluded from the analysis (per-protocol population) due to incomplete medical documentation. There were no additional exclusion criteria for patients’ participation in this study. The flow diagram of the study is shown in Supplementary Figure S1. The study was reported according to the Strengthening the Reporting of Observational Studies in Epidemiology (STROBE) recommendations (Supplementary File S1).

### 2.3. Outcomes

A primary outcome was the association of derived laboratory biomarkers (NLR, platelet-to-lymphocyte ratio, monocyte-to-lymphocyte ratio, De Ritis ratio, CRP-to-albumin ratio, LDH-to-hemoglobin ratio, CRP-to-lymphocyte ratio, RDW-to-albumin ratio, platelet-to-albumin ratio, D-Dimer-to-albumin ratio, D-Dimer-to-NLR ratio, LDH-to-albumin ratio, and LDH-to-WBC ratio) and 30-day COVID-19-associated mortality. Other outcomes included calibration and discrimination of the aforementioned derived laboratory biomarkers with regard to 30-day COVID-19-associated mortality.

### 2.4. Data Sources and Assessment

Detailed medical data, including baseline characteristics (age, sex, comorbidities, vaccination status), transplantation specifics (time since kidney transplantation, chronic and modified immunosuppressive therapy), COVID-19 details (symptoms and their duration, oxygen and specific antiviral therapy applied, outcomes), laboratory results (WBC, red blood cell count, platelet count, CRP, D-dimers level, aspartate transaminase (AST), alanine transaminase (ALT), gamma-glutamyl transferase (GGT), LDH, blood urea nitrogen (BUN), serum creatinine), and radiographic findings were obtained from the hospital’s electronic medical records by experienced physicians. Biomarkers were derived using the previously mentioned laboratory results upon hospital admission.

### 2.5. Laboratory Analysis

Blood samples were collected from all patients at the time of hospital admission. All blood samples were drawn over a polyethylene catheter from the antecubital vein and analyzed in a single laboratory by an experienced biochemist. Complete blood count (CBC) was determined by flow cytometry. Albumin levels were determined using the 2-point end colorimetric assay (Roche/Hitachi Cobas C^®^, Mannheim, Germany) with linear 2-point calibration and measuring range of 2–60 g/L (coefficient of variation 0.4–0.7%). Plasma CRP levels were determined using the 2-point end particle-enhanced immunoturbidimetric assay (Roche Cobas C^®^) with non-linear full calibration and measuring range of 0.6–350 mg/L (coefficient of variation 1.2–4.8%). CRP levels represent standard CRP values (coefficient of variation %). ALT and AST levels were analyzed according to the recommendations of the International Federation of Clinical Chemistry and Laboratory Medicine (Roche Cobas C^®^), with optimization for performance and stability, linear 2-point calibration and measuring range of 5–700 U/L (coefficient of variation 0.5–3.1% and 0.4–6.8%, respectively). LDH levels were determined according to the recommendations of the International Federation of Clinical Chemistry and Laboratory Medicine (UV-test assay; Roche/Hitachi Cobas C^®^), with linear 2-point calibration and measuring range of 10–1000 U/L (coefficient of variation 0.3–1.0%). Other biochemical analyses were measured by standard laboratory methods. All analyses underwent quality control in the laboratory settings.

### 2.6. Laboratory Derived Biomarkers

All laboratory-derived biomarkers were calculated using appropriate laboratory parameters. Their details are presented below.

The NLR is a widely used inflammatory biomarker that is associated with mortality in the overall COVID-19 population [7,8]. There are several studies on KTR with COVID-19 that showed discrepant findings [12,13,14] but warrant further analyses.

The platelet-to-lymphocyte ratio represents an easy to use biomarker that was associated with all-cause mortality in patients with end-stage kidney disease (ESRD) [15]. The platelet-to-lymphocyte ratio showed good performance in predicting the risk of severe disease in the general COVID-19 population [16], but was not evaluated in subgroups of KTR.

The monocyte-to-lymphocyte ratio was evaluated in the setting of COVID-19, showing that high monocyte-to-lymphocyte ratio at the time of hospital admission could predict in-hospital mortality in patients with CKD and COVID-19 [9], but was not evaluated in KTR.

The De Ritis ratio is a well-known marker of liver disease, but also shows a significant association with in-hospital mortality in the general COVID-19 population [10]. Furthermore, Liu et al. suggested that patients with COVID-19 and high De Ritis ratio should be carefully monitored due to increased adverse events across the long-term follow-up [17]. It was not investigated in the setting of KTR and COVID-19.

The CRP-to-albumin ratio has previously shown a good association with severe COVID-19, while its relation to mortality was not detected [18]. It was not evaluated in KTR with COVID-19.

The LDH-to-hemoglobin ratio is an under-investigated but potentially useful biomarker that is comprised of LDH as a strong predictor of severe COVID-19, and hemoglobin as a determinant of frailty and oxygen transport properties [19,20].

The CRP-to-lymphocyte ratio is a well-known predictor of adverse events in cancer population [21] but was not investigated in the setting of COVID-19.

The RDW-to-albumin ratio is associated with the higher mortality rate amongst patients treated in the Intensive Care Unit (ICU) due to acute respiratory distress syndrome (ARDS) [22] but was not evaluated in the setting of COVID-19.

The platelet-to-albumin ratio is a potential predictor of mortality in patients treated with peritoneal dialysis [23] and could be used to predict disease progression in patients with IgA nephropathy [24] but was not evaluated in the setting of COVID-19.

D-dimers are well-known markers of endothelial dysfunction and impaired prognosis in COVID-19 [25]. The D-dimer-to-albumin ratio is a novel biomarker that predicts mortality in hospitalized COVID-19 patients [11]. It was not evaluated in KTR with COVID-19.

The D-Dimer-to-NLR ratio was not evaluated in the COVID-19 population, but each component per se shows a good association with worse outcome in the general COVID-19 population [26,27,28].

The LDH-to-albumin ratio showed good performance in patients developing pneumonia after stroke, as well as in those with lower respiratory tract infections [29,30]. Furthermore, it arises as a potential risk stratification tool in the general COVID-19 population that could detect patients at risk of worse outcomes upon hospital admission [31]. Its performance was not analyzed in specific subpopulations such as KTR with COVID-19.

The LDH-to-WBC ratio has been previously studied as a part of the CROW-65 risk score in COVID-19 patients treated with high-flow nasal oxygen and KTR with COVID-19 [6,32], but it lacks comparison with other laboratory biomarkers.

### 2.7. Statistical Analysis

Statistical analysis was carried out using the Stata software (StataCorp, College Station, TX, USA; version 17). Categorical variables were expressed as numbers (percentages), and the continuous data were presented as median (interquartile range [IQR]). The Cox regression analysis (proportional hazards) was used to define the association of derived laboratory biomarkers and 30-day mortality. It was reported as hazard ratios (HR) and 95% confidence intervals (95% CI) which correspond to a 1-unit increase/decrease of each biomarker (continuous scale). The discrimination of the studied biomarkers was assessed by Harrell’s C concordance and Somers’ D index. The calibration of the models was expressed by the Hosmer–Lemeshov test, and visually by calibration plots.

The predictive accuracy of the biomarkers was further determined by the receiver operating characteristic (ROC) and area under the curve (AUC). Optimal cut-offs for each biomarker were determined by Youden’s index, while the statistical comparison of different AUC curves was conducted using a method by Hanley and McNeil [33]. A two-sided *p*-value of <0.05 was considered significant.

## 3. Results

All-cause mortality occurred in a total of 12 patients (18.5%) during the 30-day follow-up. The baseline characteristics of the study population and values of derived biomarkers are presented in Supplementary Tables S1 and S2. A significant association with the primary outcome was observed for the following biomarkers: De Ritis ratio (HR 3.83, 95% CI 1.57–9.35, *p* = 0.003), CRP-to-albumin ratio (HR 1.36, 95% CI 1.13–1-64, *p* = 0.001), LDH-to-hemoglobin ratio (HR 1.44, 95% CI 1.07–1.92, *p* = 0.015), CRP-to-lymphocyte ratio (HR 1.03, 95% CI 1.01–1.07, *p* = 0.003), D-dimer-to-albumin ratio (HR 4.94, 95% CI 1.38–7.24, *p* = 0.038), LDH-to-albumin ratio (HR 1.20, 95% CI 1.05–1.36, *p* = 0.008), and LDH-to-WBC ratio (HR 1.03 95% CI 1.01–1.05, *p* = 0.024) (Table 1).

**Figure 1:**
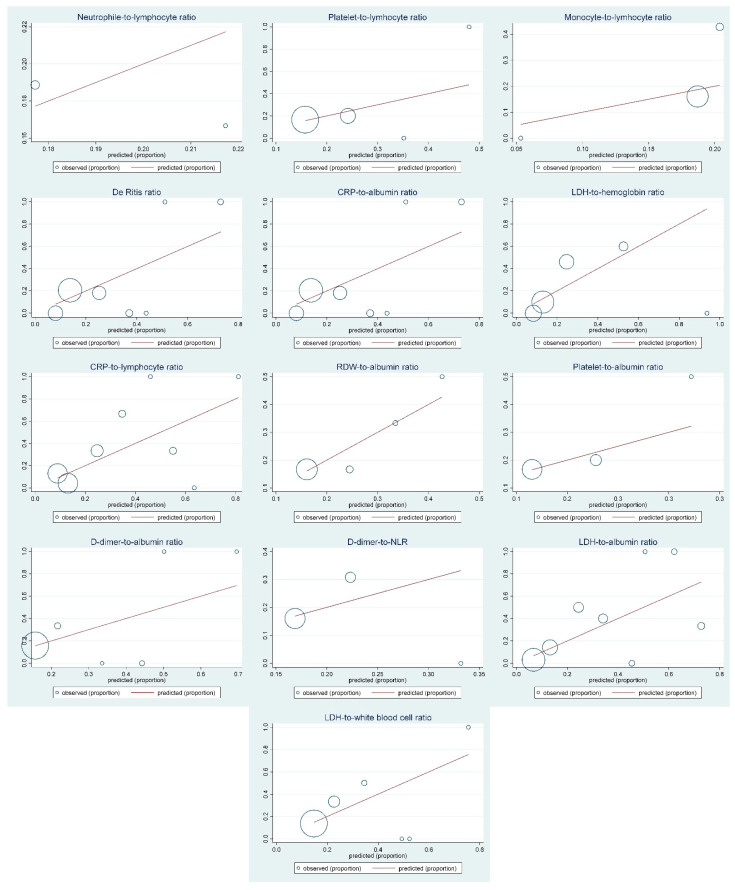
Calibration plots of the derived laboratory biomarkers. Abbreviations: CRP—C-reactive protein; CI—confidence intervals; DD—D-dimers; Hgb—hemoglobin; HR—hazard ratios; LDH—lactate dehydrogenase; MLR—monocyte-to-lymphocyte ratio; NLR—neutrophile-to-lymphocyte ratio; Plt—platelets; PLR—platelet-to-lymphocyte ratio; RDW—red cell distribution width; WBC—white blood cells.

The aforementioned biomarkers showed satisfactory discrimination in relation to the primary outcome, while the best discrimination displayed LDH-to-hemoglobin ratio (Harrell’s C 0.808 and Somers’ D 0.616) (Table 1). The overall calibration was satisfactory for all models (Table 1 and Figure 1).

**Figure 2:**
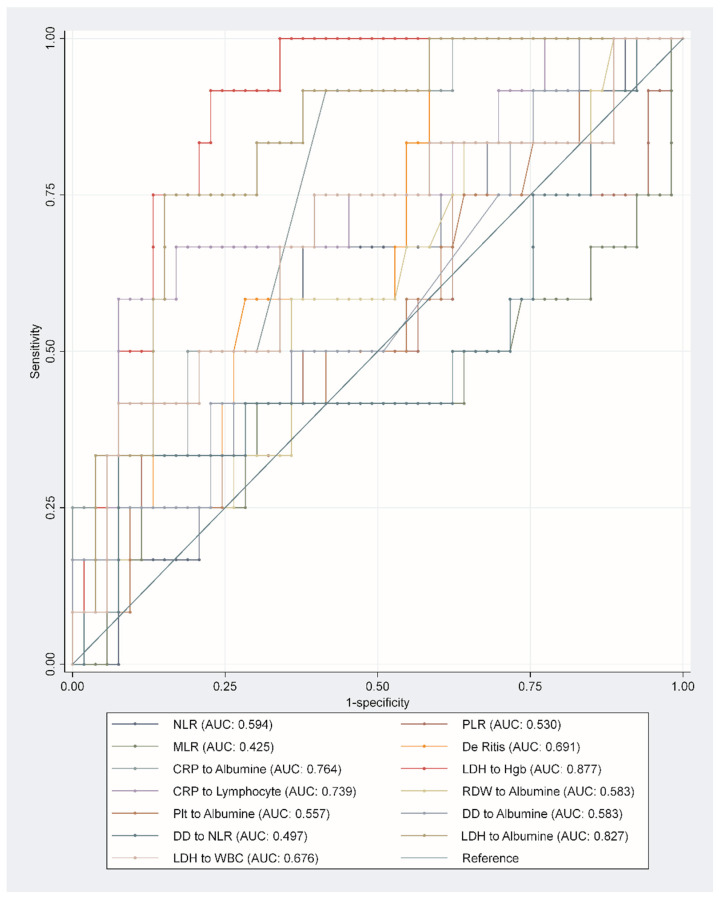
Receiver operating characteristics of the derived laboratory biomarkers. Abbreviations: CRP—C-reactive protein; CI—confidence intervals; DD—D-dimers; Hgb—hemoglobin; HR—hazard ratios; LDH—lactate dehydrogenase; MLR—monocyte-to-lymphocyte ratio; NLR—neutrophile-to-lymphocyte ratio; Plt—platelets; PLR—platelet-to-lymphocyte ratio; RDW—red cell distribution width; WBC—white blood cells.

When evaluating ROC curves of the studied biomarkers, the best values were achieved with De Ritis ratio (AUC 0.691 95% CI 0.537–0.845, *p* = 0.040), CRP-to-albumin ratio (AUC 0.764 95% CI 0.632–0.897, *p* = 0.005), LDH-to-hemoglobin ratio (AUC 0.877 95% CI 0.793–0.962, *p* < 0.001), CRP-to-lymphocyte ratio (AUC 0.739 95% CI 0.570–0.908, *p* = 0.010), and LDH-to-albumin ratio (AUC 0.827 95% CI 0.712–0.942, *p* = 0.058) (Table 2 and Figure 2), without evidence of statistically significant differences in predictive accuracy between these five laboratory biomarkers (Supplementary Table S3).

## 4. Discussion

To the best of our knowledge, this is the first study to investigate and compare the performance of different derived laboratory biomarkers with regard to 30-day mortality in KTR with COVID-19. The results of this study support several important findings. First, derived laboratory biomarkers such as the De Ritis ratio, CRP-to-albumin ratio, LDH-to-hemoglobin ratio, CRP-to-lymphocyte ratio, and LDH-to-albumin ratio showed significant association with mortality in this setting, while other biomarkers showed poor performance. Second, the best discrimination was exhibited with LDH-to-hemoglobin ratio, although its predictive accuracy for mortality did not show statistically significant difference with the four other biomarkers in the study. Finally, NLR, as the one of the most studied biomarkers, did not show good performance in assessing 30-day mortality in this setting. The high mortality rate of 18.5% in this patient population supports the need for novel and easy-to-use biomarkers/risk scores to detect patients at risk of worse outcomes in a timely fashion.

The biomarkers from this study were previously evaluated in the general COVID-19 population and their association with mortality was suggested. Specifically, platelet-to-lymphocyte ratio, CRP-to-albumin ratio and LDH-to-albumin ratio showed association with a severe disease form and worse clinical outcomes [16,18,31]. Furthermore, a significant association with increased mortality amongst the overall COVID-19 population was demonstrated for NRL, De Ritis ratio and D-dimer-to-albumin ratio, while a study on chronic kidney disease (CKD) patients revealed an association between monocyte-to-lymphocyte ratio and increased mortality [9,10,11,26,27].

The available literature lacks comprehensive analyses of the association of different derived biomarkers and mortality in KTR with COVID-19. The only biomarker that was previously evaluated in this population is NLR, but the available findings are inconsistent. A study by Peçanha-Pietrobom et al. did not determine a significant difference in admission NLR levels between KTR and their counterparts, suggesting against their predictive role [14]. On the other hand, NLR showed an independent association with mortality in KTR with community-acquired pneumonia [34], which may be the consequence of different pathophysiology and immunologic response in COVID-19. The present study consistently did not show a significant association between NLR and mortality, which was confirmed across different performance measures. This could possibly be attributed to the interaction of specific COVID-19 immunologic responses and concomitant immunosuppression in KTR, leading to rebound effects on neutrophils. Further studies should elucidate whether this biomarker could be useful in KTR, but available data do not support its use.

LDH is a ubiquitous enzyme that proved to be an important biomarker in COVID-19. Higher levels of LDH show a significant association with worse clinical outcomes in the general COVID-19 population [19]. Few studies reported statistically significant differences in the LDH levels between KTR survivors and non-survivors [35,36]. When combining LDH with albumin as a measure of nutritional status, the LDH-to-albumin ratio shows a promising prediction of mortality in KTR with COVID-19 [36]. Additionally, anemia is a strong predictor of severe disease in the overall COVID-19 population, although available studies do not suggest a difference in hemoglobin levels between KTR survivors and non-survivors with COVID-19 [20,35,36]. The present study showed a significant association of both LDH-to-hemoglobin ratio and LDH-to-albumin ratio with 30-day mortality in KTR with COVID-19, with LDH-to-hemoglobin ratio showing higher absolute AUC values, but without a statistically significant difference between them (AUC 0.877 and 0.827, respectively). Interestingly, the LDH-to-WBC ratio did not show good performance in this study, which could be attributed to the immunologic effects of KTR with COVID-19. These findings suggest an important role of LDH in KTR with COVID-19, particularly when used in conjunction with other important determinants such as hemoglobin and albumin.

Surprisingly, the D-dimer-to-albumin ratio did not show a consistently good association with mortality in this study when analyzing different performance measures, although it showed a good association with higher mortality rate in the overall COVID-19 population [11]. Available data about the predictive role of D-dimer levels per se in KTR with COVID-19 are inconsistent [35,36,37], while studies in the general COVID-19 population demonstrate its association with worse outcomes [25]. It is known that the SARS-CoV-2 virus has a high affinity for vascular endothelium, which promotes endothelial dysfunction. It remains unclear whether immunosuppression affects this interaction in KTR with COVID-19 [38].

Both CRP-to-albumin ratio and CRP-to-lymphocyte ratio showed good performance in assessing 30-day mortality in KTR with COVID-19. Although Cravedi et al. did not report a statistically significant difference in CRP levels between KTR survivors and non-survivors with COVID-19, there was a significant difference in lymphocyte level, with non-survivors having lower lymphocyte levels (*p* = 0.004) [35]. When evaluating the CRP-to-albumin ratio in the general population, there was no association with a higher mortality rate, but there was an association with worse clinical outcomes and severe forms of the disease [18].

Results from this study could help physicians in detecting those KTR with COVID-19 who may need more intensive monitoring and eventually ICU treatment. Furthermore, this study could serve as a model and encourage further studies on a bigger sample of KTR with COVID-19, which could provide a tool for risk-stratification and detection of KTR who are at risk of worse outcomes.

This study has several limitations. First, a limited sample size from a single center could affect the statistical power of the study. Other intrinsic limitations applicable to single center studies should be acknowledged, including the treatment bias and lack of external validation. Second, due to retrospective observational design, selection bias could not be eliminated. Third, an absolute number of mortality events could have affected the performance of the biomarkers. Fourth, this study evaluated only laboratory-derived biomarkers, without any other clinical parameters. Fifth, potential analytical differences between laboratories could affect the study results. Sixth, temporary variations in COVID-19-related factors (vaccination, different viral variants, specific medication) warrant further validation of these findings. Seventh, the severity of the course of COVID-19 infection could be influenced by multiple non-accountable genetic, immunological, biochemical, and clinical factors that could affect the study findings. All aforementioned constraints limit the clinical applicability of these findings and delineate the hypothesis-generating purpose of this research study, warranting further studies. Clinical applicability of the study results is dependent on its validation in a larger sample of patients.

## 5. Conclusions

In conclusion, derived laboratory biomarkers such as the De Ritis ratio, CRP-to-albumin ratio, LDH-to-hemoglobin ratio, CRP-to-lymphocyte ratio, and LDH-to-albumin ratio show significant association with all-cause mortality in KTR with COVID-19, suggesting their potential risk stratification role. The best discrimination was exhibited with LDH-to-hemoglobin ratio. Further powered, longitudinal and multicentric studies in a larger sample of patients should investigate the clinical applicability of these hypothesis-generating findings.

## Figures and Tables

**Table 1:** Association of derived laboratory biomarkers with 30-day mortality (Cox proportional hazards logistic regression analysis).

Biomarkers	30-Day Mortality			
	HR (95% CI), *p*-Value *	Harrell’s C Concordance Index	Somers’ D Index	Hosmer–Lemeshow Test, *p*-Value
Neutrophile-to-lymphocyte ratio	1.02 (0.94–1.12), *p* = 0.634	0.597	0.194	6.00, *p* = 0.815
Platelet-to-lymphocyte ratio	1.00 (0.99–1.01), *p* = 0.086	0.580	0.160	12.53, *p* = 0.251
Monocyte-to-lymphocyte ratio	0.88 (0.43–1.81), *p* = 0.724	0.537	0.074	10.18, *p* = 0.425
De Ritis ratio	3.83 (1.57–9.35), *p* = 0.003	0.691	0.382	12.65, *p* = 0.244
CRP-to-albumin ratio	1.36 (1.13–1.64), *p* = 0.001	0.733	0.466	9.40, *p* = 0.401
LDH-to-hemoglobin ratio	1.44 (1.07–1.92), *p* = 0.015	0.808	0.616	8.38, *p* = 0.592
CRP-to-lymphocyte ratio	1.03 (1.01–1.07), *p* = 0.003	0.681	0.361	7.19, *p* = 0.707
RDW-to-albumin ratio	7.44 (0.31–11.62), *p* = 0.601	0.525	0.049	6.94, *p* = 0.731
Platelet-to-albumin ratio	1.08 (0.82–1.42), *p* = 0.597	0.540	0.080	4.74, *p* = 0.908
D-Dimer-to-albumin ratio	4.94 (1.38–7.24), *p* = 0.038	0.571	0.143	6.02, *p* = 0.734
D-Dimer-to-NLR	1.05 (0.67–1.65), *p* = 0.821	0.504	0.008	7.45, *p* = 0.682
LDH-to-albumin ratio	1.20 (1.05–1.36), *p* = 0.008	0.751	0.502	7.07, *p* = 0.719
LDH-to-WBC ratio	1.03 (1.01–1.05), *p* = 0.024	0.629	0.259	8.94, *p* = 0.538

* Cox proportional hazards regression analysis (univariate model). Abbreviations: CRP—C-reactive protein; CI—confidence intervals; HR—hazard ratios; LDH—lactate dehydrogenase; NLR—neutrophile-to-lymphocyte ratio; RDW—red cell distribution width; WBC—white blood cells.

**Table 2:** ROC curve analysis of derived laboratory biomarkers with 30-day mortality.

Biomarkers	C-Statistic (95% CI)	*p*-Value	Sensitivity/Specificity	Youden’s Index
Neutrophile-to-lymphocyte ratio	0.594 (0.420–0.768)	0.310	0.67/0.62	>6.17
Platelet-to-lymphocyte ratio	0.530 (0.321–0.739)	0.748	0.50/0.62	>290.84
Monocyte-to-lymphocyte ratio	0.425 (0.209–0.642)	0.422	0.42/0.70	>0.59
De Ritis ratio	0.691 (0.537–0.845)	0.040	0.58/0.72	>1.48
CRP-to-albumin ratio	0.764 (0.632–0.897)	0.005	0.92/0.58	>2.06
LDH-to-hemoglobin ratio	0.877 (0.793–0.962)	<0.001	0.92/0.77	>2.21
CRP-to-lymphocyte ratio	0.739 (0.570–0.908)	0.010	0.67/0.70	>77.80
RDW-to-albumin ratio	0.583 (0.405–0.761)	0.370	0.58/0.64	>0.42
Platelet-to-albumin ratio	0.557 (0.376–0.737)	0.543	0.42/0.72	>6.79
D-Dimer-to-albumin ratio	0.583 (0.400–0.766)	0.370	0.42/0.77	>0.04
D-Dimer-to-NLR	0.497 (0.288–0.706)	0.973	0.33/0.92	>0.39
LDH-to-albumin ratio	0.827 (0.712–0.942)	<0.001	0.75/0.85	>10.78
LDH-to-WBC ratio	0.676 (0.492–0.860)	0.058	0.75/0.60	>43.13

Abbreviations: CRP—C-reactive protein; CI—confidence intervals; HR—hazard ratios; LDH—lactate dehydrogenase; NLR—neutrophile-to-lymphocyte ratio; RDW—red cell distribution width; WBC—white blood cells.

## Data Availability

The data that support the findings of this study are available on request from the corresponding author [A.M.].

## Supplementary Materials

The following supporting information can be downloaded at: https://www.mdpi.com/article/10.3390/life12122068/s1, Supplementary Table S1: Baseline characteristics of the study sample; Supplementary Table S2: Descriptive statistics of the derived laboratory biomarkers from the study; Supplementary Table S3: Comparison of ROC curves between the derived laboratory biomarkers from the study; Supplementary Figure S1: Flow diagram; Supplementary File S1: STROBE checklist of the study.
