# Supplementary material for: Performance of Derived Laboratory Biomarkers with Regard to 30-Day Mortality in Kidney Transplant Recipients with COVID-19
Source: Life (Basel). 2022 Dec 9;12(12):2068. doi: 10.3390/life12122068 (PMC9787399; doi:10.3390/life12122068)

SUPPLEMENTARY FILES

Supplementary Table S1. Baseline characteristics of the study sample.

Variables	Study sample (N=65)
**Age (years)**	57 (45-66)
**Female sex**	43 (66.2%)
**Transplant duration (years)**	8 (2-11)
**Any vaccination dose**	14 (21.5%)
**Symptom duration upon hospital admission (days)**	7 (3-11)
**Comorbidities**	
**Arterial hypertension**	53 (81.5%)
**Diabetes mellitus**	18 (27.7%)
**Chronic heart failure**	5 (7.7%)
**Active smoking**	5 (7.7%)
**Atrial fibrillation**	7 (10.8%)
**Prior AMI**	7 (10.8%)
**Prior CVI**	6 (9.2%)
**PAD**	10 (15.4%)
**COPD/asthma**	4 (6.2%)
**Chronic immunosuppressive therapy**	
**Mycophenolate Mofetil**	58 (89.2%)
**Azathioprine**	1 (1.5%)
**Cyclosporine**	20 (30.8%)
**Tacrolimus**	36 (55.4%)
**Everolimus**	8 (12.3%)
**Sirolimus**	3 (4.6%)
**Prednisone**	65 (100.0%)
**COVID-19 related therapy**	
**Reconvalescent plasma**	6 (9.2%)
**Casirivimab/Imdevimab**	2 (3.1%)
**Remdesivir**	44 (67.7%)
**Oxygen therapy**	24 (36.9%)

Data are expressed as number (percent) or median (interquartile range).
Abbreviations: AMI – acute myocardial infarction; COPD – chronic obstructive pulmonary disease; CVI – cerebrovascular incident; PAD – peripheral arterial disease.

Supplementary Table S2. Descriptive statistics of the derived laboratory biomarkers from the study.

Biomarkers	Study sample (N=65)
**Neutrophile-to-lymphocyte ratio**	5.55 (4.28-10.85)
**Platelet-to-lymphocyte ratio**	271.00 (183.67-395.35)
**Monocyte-to-lymphocyte ratio**	0.48 (0.37-0.73)
**De Ritis ratio**	1.31 (1.04-1.69)
**CRP-to-albumin ratio**	2.06 (0.82-2.82)
**LDH-to-hemoglobin ratio**	1.95 (1.53-2.84)
**CRP-to-lymphocyte ratio**	56.80 (22.19-124.53)
**RDW-to-albumin ratio**	0.41 (0.38-0.44)
**Platelet-to-albumin ratio**	5.63 (4.69-7.05)
**D-Dimer-to-albumin ratio**	0.02 (0.02-0.04)
**D-Dimer-to-NLR ratio**	0.15 (0.09-0.23)
**LDH-to-albumin ratio**	7.36 (5.82-10.86)
**LDH-to-WBC ratio**	41.03 (30.18-53.73)

Data are expressed as median (interquartile range).
Abbreviations: CRP – C-reactive protein; DD – D-dimers; Hgb – hemoglobin; HR – hazard ratios; LDH – lactate dehydrogenase; MLR – monocyte-to-lymphocyte ratio; NLR – neutrophile-to-lymphocyte ratio; Plt – platelets; PLR – platelet-to-lymphocyte ratio; RDW – red cell distribution width; WBC – white blood cells.

Supplementary Table S3. Comparison of ROC curves between the derived laboratory biomarkers from the study.

Variables	Neutrophile-to-lymphocyte ratio	Platelet-to-lymphocyte ratio	Monocyte-to-lymphocyte ratio	De Ritis ratio	CRP-to-albumin ratio	LDH-to-hemoglobin ratio	CRP-to-lymphocyte ratio	RDW-to-albumin ratio	Platelet-to-albumin ratio	D-Dimer-to-albumin ratio	D-Dimer-to-NLR ratio	LDH-to-albumin ratio	LDH-to-WBC ratio
**Neutrophile-to-lymphocyte ratio**		0.065<br>p=0.342	0.020<br>p=0.845	0.097<br>p=0.325	0.170<br>p=0.095	0.283<br>p<0.001	0.145<br>p=0.088	0.008<br>p=0.944	0.038<br>p=0.767	0.009<br>p=0.941	0.091<br>p=0.343	0.233<br>p=0.006	0.082<br>p=0.494
**Platelet-to-lymphocyte ratio**			0.044<br>p=0.713	0.161<br>p=0.155	0.234<br>p=0.047	0.347<br>p<0.001	0.209<br>p=0.012	0.057<br>p=0.667	0.027<br>p=0.831	0.056<br>p=0.650	0.027<br>p=0.827	0.297<br>p=0.005	0.146<br>p=0.189
**Monocyte-to-lymphocyte ratio**				0.117<br>p=0.256	0.190<br>p=0.128	0.303<br>p=0.008	0.165<br>p=0.213	0.013<br>p=0.924	0.017<br>p=0.899	0.012<br>p=0.923	0.071<br>p=0.521	0.253<br>p=0.027	0.102<br>p=0.455
**De Ritis ratio**					0.073<br>p=0.459	0.186<br>p=0.021	0.048<br>p=0.660	0.105<br>p=0.357	0.134<br>p=0.251	0.105<br>p=0.351	0.188<br>p=0.131	0.136<br>p=0.114	0.015<br>p=0.899
**CRP-to-albumin ratio**						0.113<br>p=0.082	0.025<br>p=710	0.178<br>p=0.096	0.208<br>p=0.052	0.178<br>p=0.091	0.261<br>p=0.021	0.063<br>p=0.355	0.088<br>p=0.389
**LDH-to-hemoglobin ratio**							0.138<br>p=0.064	0.291<br>p=0.001	0.321<br>p=0.001	0.292<br>p<0.001	0.374<br>p<0.001	0.050<br>p=0.080	0.201<br>p=0.014
**CRP-to-lymphocyte ratio**								0.153<br>p=0.188	0.182<br>p=0.111	0.153<br>p=0.166	0.236<br>p=0.061	0.088<br>p=266	0.063<br>p=0.476
**RDW-to-albumin ratio**									0.030<br>p=0.766	0.001<br>p=0.994	0.083<br>p=0.523	0.241<br>p=0.002	0.090<br>p=0.481
**Platelet-to-albumin ratio**										0.029<br>p=0.778	0.054<br>p=0.649	0.270<br>p=0.004	0.119<br>p=0.261
**D-Dimer-to-albumin ratio**											0.083<br>p=0.357	0.241<br>p=0.003	0.090<br>p=0.463
**D-Dimer-to-NLR ratio**												0.324<br>p=0.003	0.173<br>p=0.216
**LDH-to-albumin ratio**													0.151<br>p=0.095
**LDH-to-WBC ratio**													

*Data are presented as the difference between areas - method by *Hanley & McNeil* (1983).

Abbreviations: CRP – C-reactive protein; CI – confidence intervals; DD – D-dimers; Hgb – hemoglobin; HR – hazard ratios; LDH – lactate dehydrogenase; MLR – monocyte-to-lymphocyte ratio; NLR – neutrophile-to-lymphocyte ratio; Plt – platelets; PLR – platelet-to-lymphocyte ratio; RDW – red cell distribution width; WBC – white blood cells.

Supplementary Figure S1. Flow diagram.

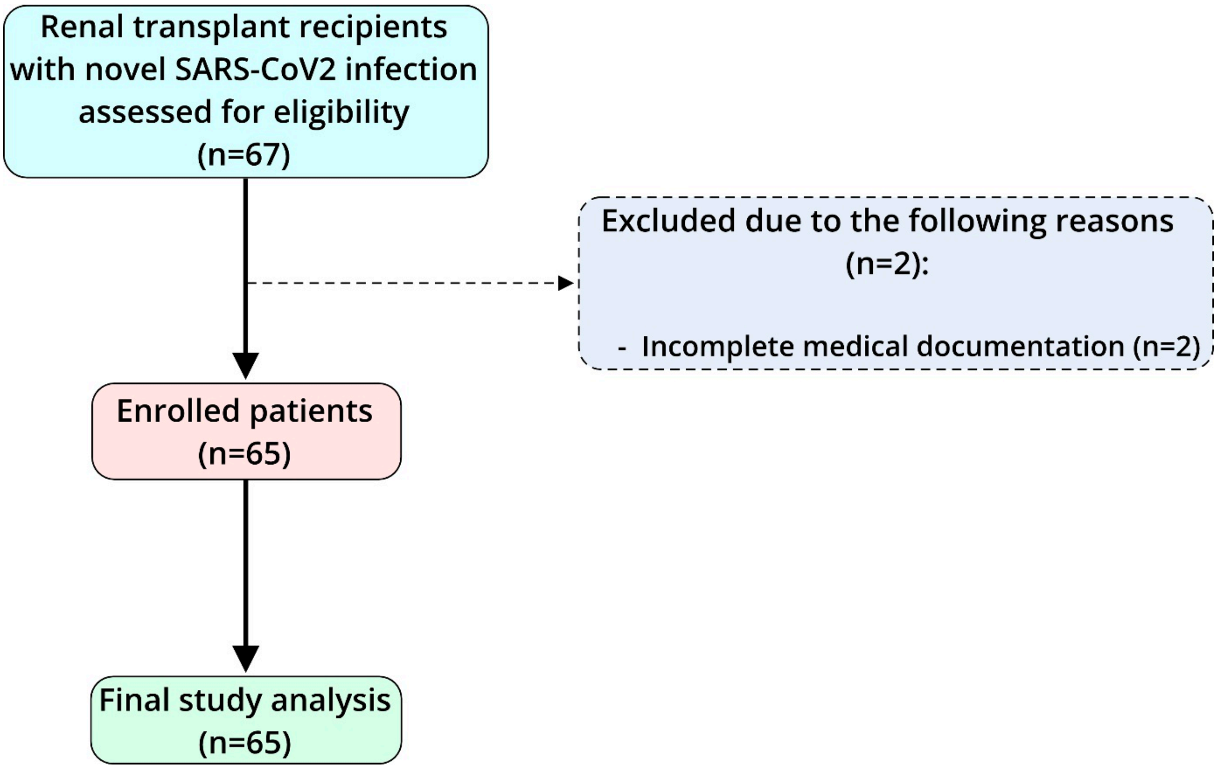

Supplement: Supplementary file 1 [file life-12-02068-s001.zip › life-1983033-supplementary tables and figures.pdf]
